# Supplementary material for: Heat adaptation of phage T7 under an extended genetic code
Source: Virus Evol. 2021 Dec 1;7(2):veab100. doi: 10.1093/ve/veab100 (PMC8923235; doi:10.1093/ve/veab100)
Supplement: veab100_Supp [file veab100_supp.zip › Sequencing_Analysis.zip!/Sequencing_Analysis/S1/evidence/SNP_3.html]

BRESEQ :: Evidence


| Predicted mutation | | | | | | |
| --- | --- | --- | --- | --- | --- | --- |
| evidence | seq id | position | mutation | annotation | gene | description |
| RA | NC\_001604.1 | 15,094 | G→T | intergenic (–/–) | – / – | –/– |

| Read alignment evidence... | | | | | | | | | | | |
| --- | --- | --- | --- | --- | --- | --- | --- | --- | --- | --- | --- |
|  | seq id | position | | ref | new | freq | score (cons/poly) | reads | annotation | genes | product |
| \* | NC\_001604.1 | 15,094 | 0 | G | T | 99.1% | 11041.6 / NA | 4448 | intergenic (–/–) | *–/–* | –/– |
| Reads supporting (aligned to +/- strand):  **ref** base G (7/33);  **new** base T (2221/2170);  **total** (2232/2216) | | | | | | | | | | | |
| Fisher's exact test for biased strand distribution *p*-value = 2.14e-05 | | | | | | | | | | | |
| Kolmogorov-Smirnov test that lower quality scores support variant *p*-value = 1.00e+00 | | | | | | | | | | | |

Only 100 of 4477 total aligned reads displayed.  

`AGAGCAGGGTGAAGAATACGTTGACGGAATGGAGTGGTGGAACTTCAACGAAGAGATGATGGACTATAACGTTCAGGACGTTGTGGTAACTAAAGCTCTCCTTGAGAAGCTACTCTCTGACAAACATTACTTCCCTCCTGAGATTGACTTTACGGACGTAGGATACACTACGTTCTGGTCAGAATCCCTTGAGGCCGTTGACATTGAACATCGTGCTGCATGGCTGCTCGCTAAACAAGAGCGCAACGGGTTCCCGTTTGACACAAAAGCAATCGAAGAGTTGTACGTAGAGTTAGCTGCTCGCCGCTCTGAGTTGCTCCGTAAATTGACCGAAACGTTCGGCTCGTGGTATCAGCCTAAAGGTGGCACTGAGATGTTCTGCCATCCGCGAACAGGTAAGCCACTACCTAAATACCCTCGCATTAAGACACCTAAAGTTGGTGGTATCTTTAAGAAGCCTAAGAACAAGGCACAGCGAGAAGGCCGTGAGCCTTGCGAACTTGATACCCGCGAGTACGTTGCTGGTGCTCCTTACACCCCAGTTGAACATGTTGTGTTTAACCCTTCGTCTCGTGACCACATTCAGAAGAAACTCC  >  NC_001604.1/14796‑15391`  

                                                                                                                                                                                                                                                                                                          `|`                                                                                                                                                                                                                                                                                                           
      `gggTGAAGAATACGTTGACGGAATGGAGTGGTGGAACTTCAACGAAGAGATGATGGACTATAACGTTCAGGACGTTGTGGTAACTAAAGCTCTCCTTGAGAAGCTACTCTCTGACAAACATTACTTCCCTCCTGAGATTGACTTTACGGACGTAGGATACACTACGTTCTGGTCAGAATCCCTTGAGGCCGTTGACATTGAACATCGTGCTGCATGGCTGCTCGCTAAACAAGAGCGCAACGGGTTCCCGTTTGACCAAAAAGCAATCGAAGAGTTGTACGTAGAATTCGCTTCTagccgc                                                                                                                                                                                                                                                                                                   >  1:670104/1‑301 (MQ=255)`  
          `gaagaaTACGTTGACGGAATGGAGTGGTGGAACTTCAACGAAGAGATGATGGACTATAACGTTCAGGACGTTGTGGTAACTAAAGCTCTCCTTGAGAAGCTACTCTCTGACAAACATTACTTCCCTCCTGAGATTGACTTTACGGACGTAGGATCCACTACGTTCTGGTCAGAATCCCTTGAGGCCGTTGACATTGAAAATCGTGCTGCATGGCTGCTCGCTAAACAAGAGCGCAACGGGTTCCCGTTTGACACAAAAGCAATCGAAGGGTTGTACGTAGAGTTAGCTTCTCGCCGCTCTg                                                                                                                                                                                                                                                                                               >  1:43950/1‑301 (MQ=255)`  
             `gaaTACGTTGACGGAATGGAGTGGTGGAACTTCAACGAAGAGATGATGGACTATAACGTTCAGGACGTTGTGGTAACTAAAGCTCTCCTTGAGAAGCTACTCTCTGACAAACATTACTTCCCTCCTGAGATTGACTTTACGGACGTAGGATACACTACGTTCTGGTCAGAATCCCTTGAGGCCGTTGACATTGAACATCGTGCTGCATGGCTGCTCGCTAAACAAGAGCGCAACGGGTTCCCGTTTGACACAAAAGCAATCGAAGAGTTGTACGTAGAGTTAGCTTCTCGCCGCTCTGAGt                                                                                                                                                                                                                                                                                            <  1:357070/301‑1 (MQ=255)`  
                  `cGTTGACGGAATGGAGTGGTGGAACTTCAACGAAGATATGATGGACTATAACGTTCAGGACGTTGTGGTAACTAAAGCTCTCCTTGAGAAGCTACTCTCTGACAAACATTACTTCCCTCCTGAGATTGACTTTACGGACGTAGGATACACTACGTTCTGGTCAGAATCCCTTGAGGCCGTTGACATTGAACATCGTGCTGCATGGCTGCTCGCTAAACAAGAGCGCAACGGGTTCCCGTTTGACACAAAAGCAATCGAAGAGTTGTACGTAGAGTTAGCTTCTCGCCGCTCTGAGTTGCTc                                                                                                                                                                                                                                                                                       <  1:194147/301‑1 (MQ=255)`  
                        `cGGAATGGAGTGGTGGAACTTCAACGAAGAGATGATGGACTATAACGTTCAGGACGTTGTGGTAACTAAAGCTCTCCTTGAGAAGCTACTCTCTGACAAACATTACTTCCCTCCTGAGATTGACTTTACGGACGTAGGATACACTACGTTCTGGTCAGAATCCCTTTAGGCCGTTGACATTTAACATCGTGCTGCATGGCTGCTCGCTAAACAAGAGCGCAACGGGTTCCCGTTTGACACAAAAGCAATCGAAGGGTTGTACGTAGAGTTAGCTTCTCGCCGCTCTGAGTTGCGCAGTaca                                                                                                                                                                                                                                                                                 >  1:351304/1‑299 (MQ=255)`  
                          `gAATGGAGTGGTGGAACTTCAACGAAGAGATGATGGACTATAACGTTCAGGACGTTGTGGTAACTAAAGCTCTCCTTGAGAAGCTACTCTCTGACAAACATTACTTCCCTCCTGAGATTGACTTTACGGACGTAGGATACACTACGTTCTGGTCAGAATCCCTTGAGGCCGTTGACATTGAACATCGTGCTGCATGGCTGCTCGCTAAACAAGAGCGCAACGGGTTCCCGTTTGACACAAAAGCAATCGAAGAGTTGTACGTAGAGTTAGCTTCTCGCCGCTCTGAGTTGCTcctgtctct                                                                                                                                                                                                                                                                               >  1:132487/1‑294 (MQ=255)`  
                          `aagagacagggtggAACTTCAACGAAGAGATGATGGACTATAACGTTCAGGACGTTGTGGTAACTAAAGCTCTCCTTGAGAAGCTACTCTCTGACAAACATTACTTCCCTCCTGAGATTGACTTTACGGACGTAGGATACACTACGTTCTGGTCAGAATCCCTTGAGGCCGTTGACATTGAACATCGTGCTGCATGGCTGCTCGCTAAACAAGAGCGCAACGGGTTCCCGTTTGACACAAAAGCAATCGAAGAGTTGTACGTAGAGTTAGCTTCTCGCCGCTCTGAGTTGCTCCGTAAAtt                                                                                                                                                                                                                                                                               <  1:498665/292‑1 (MQ=255)`  
                               `gAGTGGTGGAACTTCAACGAAGAGATGATGGACTATAACGTTCAGGACGTTGTGGTAACTAAAGCTCTCCTTGAGAAGCTACTCTCTGACAAACATTACTTCCCTCCTGAGATTGACTTTACGGACGTAGGATACACTACGTTCTGGTCAGAATCCCTTGAGGCCGTTGACATTGAACATCGTGCTGCATGGCTGCTCGCTAAACAAGAGCGCAACGGGTTCCCGTTTGACACAAAAGCAATCGAAGAGTTGTACGTAGAGTTAGCTTCTCGCCGCTCTGCGtctgtctcttatactaatc                                                                                                                                                                                                                                                                          >  1:533601/1‑283 (MQ=255)`  
                                    `gtggAACTTCAACGAAGAGATGATGGACTATAACGTTCAGGACGTTGTGGTAACTAAAGCTCTCCTTGAGAAGCTACTCTCTGACAAACATTACTTCCCTCCTGAGATTGACTTTACGGACGTAGGATACACTACGTTCTGGTCAGAATCCCTTGAGGCCGTTGACTTTGAACATCGTGCTGCATGGCTGCTCGCTCAACAAGAGCGCAACGGGTTCCCGTTTGACACAAAAGCAATCGAAGAGTTGTACGTCGAGTTAGCTTCTCGCCGCTCTGAGTTTCTCCGTAAATTGACCGAAACg                                                                                                                                                                                                                                                                     >  1:165245/1‑301 (MQ=255)`  
                                    `gtggAACTTCAACGAAGAGATGATGGACTATAACGTTCAGGACGTTGTGGTAACTAAAGCTCTCCTTGAGAAGCTACTCTCTGACAAACATTACTTCCCTCCTGAGATTGACTTTACGGACGTAGGATACACTACGATCTGGTCAGAATCCCTTGAGGCCGTTGACATTGAACATCGTGCTGCATGGCTGCTCGCTAAACAAGAGCGCAACGGGTTCCCGTTTGACACAAAAGCAATCGAAGAGTTGTACGTAGAGTTAGCTTCTCGCCGCCCTGAGTTGCTCCGTCACTTGACCGAAACg                                                                                                                                                                                                                                                                     >  1:70240/1‑301 (MQ=255)`  
                                            `tCAACGAAGAGATGATGGACTATAACGTTCAGGACGTTGTGGTAACTAAAGCTCTCCTTGAGAAGCTACTCTCTGACAAACATTACTTCCCTCCTGAGATTGACTTTACGGACGTAGGATCCACTACGTTCTGGTCAGAATCCCTTTAGGCCGTTGACATTGAACATCCTGCTGCATGGCTGCTCGCTAAACAAGAGCGCAACGGGGTCCCGTTTGACACAAAAGCAATCGAAGAGTTGTACGTAGAGTTAGCTTCTCGCCGCACTGATTTGCTCCGTAAATTTACCGAAACGTTCGGccc                                                                                                                                                                                                                                                             >  1:641091/1‑299 (MQ=255)`  
                                                `gtgtataagagcccgcTATAACGTTCAGGACGTTGTGATAACTATAGCTCTCATTGAGAAGCTACTCTCTGACAAACATTACTTCCCTCCTGAGATTGACTTTACGGACGTAGGATACACTACTTTCTGGTCAGAATCCCTTGAGGCCGTTGACATTGAACATCGTGCTGCATGGCTGCTCGCTAAACAAGAGCGCAACGGGTTCCCGTTTGACACAAAAGCAATCGAAGAGTTGTACGTAGAGTTATCTTCTCGCCACTCTGATTTGCTCCGTAAATTGACCGAAACGTTCGGCTCGTgg                                                                                                                                                                                                                                                         <  1:304515/286‑1 (MQ=255)`  
                                                 `gAAGAGATGATTGACTATAACGTTCAGGACGTTTTGGTAACTACAGCTCTCCTTGAGAAGATACTCTCTGACAAACATTACTTCCCTCCTGAGATTGACTTTACGGACGTAGGATACACTACGTTCTGGTCAGAATCCCTTGAGGCCGTTGACATTGAACATCGTGCTGCATGGCTGCTCGCTAAACAAGAGCGCAACGGGTTCCCGTTTGACACAAAAGCAATCTAAGAGTTGTACGTAGAGTTAGCTTCTCGCCGCTCTGAGTTGCTCCGTAAATTGACCGAAACGTTCGGCTCGTGGt                                                                                                                                                                                                                                                        <  1:411176/301‑1 (MQ=255)`  
                                                   `agagATGATGGACTATAACGTTCAGGACGTTGTGGTAACTAAAGCTCGCCTTGAGAAGCTACGCTCTGACAAACATTACTTCCCGCCTGAGATTGACTTTACGGACGTAGGATACACTACGTTCTGGTCAGAATCCCTTGAGGCCGTTGACATTGAACATCGTGCTGCATGGCTGCTCGCTAAACAAGAGCGCAACGGGTTCCCGTTTGACACAAAAGCAATCGAAGAGTTGTACGTAGAGTTAGCTTCTCGCCGCTCTGAGTTGCTCCGTAAATTGACCGAAACGTTCGGCTCGTGGTAt                                                                                                                                                                                                                                                      <  1:563274/301‑1 (MQ=255)`  
                                                      `gatgatGGACTATAACGTTCAGGACGTTGTGGTAACTAAAGCTCTCCTTGAGAAGCTACTCTCTGACAAACATTACTTCCCTCCTGAGATTGACTTTACGGACGTAGGATACACTACGTTCTGGTCAGAATCCCTTGAGGCCGTTGACATTGAACATCGTGCTGCATGGCTGCTCGCTAAACAAGAGCGCAACGGGTTCCCGTTTGACACAAAAGCAATCGAAGAGTTGTACGTAGAGTTAGCTTCTCGCCGCTCTGAGTTGCTCCGTAAATTGACCGAAACGTTCGGCTCGTGGTATCAg                                                                                                                                                                                                                                                   >  1:600937/1‑301 (MQ=255)`  
                                                      `gatgatGGACTATAACGTTCAGGACGTTGTGGTAACTAAAGCTCTCCTTGAGAAGCTACTCTCTGACAAACATTACTTCCCTCCTGAGATTGACTTTACGGACGTAGGATACACTACGCTCTGGTCAGAATCCCTTGAGGCCGTTGACATTGAACATCGTGCTGCATGACTGCTCGCTAAACAAGAGCGCAACGGGTTCCCGTTTGACACAAAAGCAATCGAAGAGTTGTACGTAGAGTTAGCTTCTCGCCGCTCTGAGTTGCTCCGTACATTGACCGAAACGTTCGGCTCGTGGTATCAg                                                                                                                                                                                                                                                   >  1:551520/1‑301 (MQ=255)`  
                                                         `gatgGACTATAACGTTCAGGACGTTGTGGTAACTAAAGCTCTCCTTGAGAAGCTACTCTCTGACAAACATTACTTCCCTCCTGAGATTGACTTTACGGACGTAGGATACACTACGTTCTGGTCAGAATCCCTTGAGGCCGTTGACATTGAACATCGTGCTGCATGGCTGCTCGCTAAACAAGAGCGCAACGGGTTCCCGTTTGACACAAAAGCAATCGAAGAGTTGTACGTAGAGTTAGCTTCTCGCCCCTCTGAGTTGCTCCGTAAATTGACCGAAACGTTGGGCTCGTGGTATCAGCCt                                                                                                                                                                                                                                                >  1:50411/1‑301 (MQ=255)`  
                                                            `ggACTATAACGTTCAGGACGTTGTGGTAACTAAAGCTCTCCTTGAGAAGCTACTCTCTGACAAACATTACTTCCCTCCTGAGATTGACTTTACGGACGTAGGATACACTACGTTCTGGTCAGAATCCCTTGAGGCCGTTGACATTGAACATCGTGCTGCATGGCTGCTCGCTAAACAAGAGCGCAACGGGTTCCCGTTTGACACAACAGCAATCGAAGAGTTGTACGTAGAGTTAGCTTCTCGCCGCTCTGAGTTGCTCCGTAAATTGACCGAAACGTTCGGCTCGTGGTATCAGCCTaaa                                                                                                                                                                                                                                             >  1:210605/1‑301 (MQ=255)`  
                                                            `ggACTATAACGTTCAGGACGTTGTGGTAACTAAAGCTCTCCTTGAGAAGCTACTCTCTGACAAACATTACTTCCCTCCTGAGATTGACTTTACGGACGTAGGATACACTACGTTCTGGTCAGAATCCCTTGAGGCCGTTGACATTGAACATCGTGCTGCATGGCTGCTCGCTAAACAAGAGCGCAACGGGTTCCCGTTTGACACAAAAGCAATCGAAGAGTTGTACGTAGAGTTAGCTTCTCGCCGCTCTGAGTTGCTCCGTAAATTGACCGAAACGTTCGGCTCGTGGTATCAGACTaaa                                                                                                                                                                                                                                             >  1:339960/1‑301 (MQ=255)`  
                                                             `gACTATAACGTTCAGGACGTTGTGGTAACTAAAGCTCTCCTTGAGAAGCTACTCTCTGACAAACATTACTTCCCTCCTGAGATTGACTTTACGGACGTAGGATACACTACGTTCTGGTCAGAATCCCTTGAGGCCGTTGACATTGAACATCGTGCTGCATGGCTGCTCGCTAAACAAGAGCGCAACGGGTTCCCGTTTGACACAAAAGCAATCGAAGAGTTGTACGTAGAGTTAGCTTCTCGCCGCTCTGAGTTGCTCCGTAAATTGACCGAAACGTTCGGCTctgtctcttatacacatt                                                                                                                                                                                                                                            >  1:261323/1‑284 (MQ=255)`  
                                                              `aCTATAACGTTCAGGACGTTGTGGTAACTAAAGCTCTCCTTGAGAAGCTACTCTCTGACAAACATTACTTCCCTCCTGAGATTGACTTTACGGACGTAGGATACACTACGTTCTGGTCAGAATCCCTTGAGGCCGTTGACATTGAACATCGTGCTGCATGGCTGCTCGCTAAACAAGAGCGCAACGGGTTCCCGTTTGACACAAAAGCAATCGAAGAGTTGTACGTAGAGTTAGCTTCTCGCCGCTCTGAGTTGCTCCGTAAATTGACCGAAACGTTCGGCTCGTGGTATCAGCCTAAAgg                                                                                                                                                                                                                                           >  1:664853/1‑301 (MQ=255)`  
                                                              `aCTATAACGTTCAGGACGTTGTGGTAACTAAAGCTCTCCTTGAGAAGCTACTCTCTGACAAACATTACGTCCCTCCTGAGATTGACTTTACGGACGTAGGATACACTACGTTCTGGTCAGAATCCCTTGAGGCCGTTGACATTGAACATCGTGCTGCATGGCTGCTCGCTAAACAAGAGCGCAACGGGTTCCCGTTTGACACAAAAGCAATCGAAGAGTTGTACGTAGAGTTAGCTTCTCGCCGCTCTGAGTTGCTCCGTAAATTGACCGAAACGTTCGGCTCGTGGTATCAGCCTAAAgg                                                                                                                                                                                                                                           <  1:4806/301‑1 (MQ=255)`  
                                                              `aCTATAACGTTCAGGACGTCGTTGTAACTAAAGCGCGCCTGTATAAGCTACTCTCTTACAAACATTACTTCCCTCCTGAGATTGACTTTACGGACGTAGGATACACTACGTTCTGGTCAGAATCCCTTGAGGCCGTTGACATTGAACATCGTGCTGCATGGCTGCTCGCTAAACAAGAGCGCAACGGGTTCCCGTTTGACACAAAAGCAATCGAAGAGTTGTACGTAGAGTTAGCTTCTCGCCGCTCTGAGTTGCTCCGTAAATTGACCGAAACGTTCGGCTCGTGGTATCAGCCTAAAgg                                                                                                                                                                                                                                           <  1:110509/301‑1 (MQ=255)`  
                                                               `cTATAACGTTCAGGACGTTGTGGTAACTAAAGCTCTCCTTGAGAAGATACTCTCTGACAAACATTACTTCCCTCCTCAGATTGACTTTACCGACGTAGCATACACTACGCTCTGGTCAGAATCCCTTGAGGCCCTTGACATTGAACATCGTGCTGCATGGCTGCTCGCTAAACAAGAGCGCAACGGGTTCCCGTTTGACACAAAAGCAATCGAAGAGTTGTACGTAGATGTAGCCTCTCGCCTCCCTGGATTTCTTCGTAAATTGACCTAAACGGTCGGCTCGTGGTATCAGACTTAAGGt                                                                                                                                                                                                                                          >  1:364229/1‑301 (MQ=255)`  
                                                                 `ataACGTTCAGGACGTTGTGGTAACTAAAGCTCTCCTTGAGAAGCTACTCTCTGACAAACATTACTTCCTTCCTGAGATTGACTTTACGGACGTAGGATACACTACGTTCTGGTCAGAATCCCTTGAGGCCGTTGACATTGAACATCGTGCTGCATGGCTGCTCGCTAAACAAGAGCGCAACGGGTTCCCGTTTGACACAAAAGCAATCGAAGAGTTGTACGTAGAGTTAGCTTCTCGCCGCTCTGAGTTGCTCCGTAAATTGACCGAAACGTTCGGCTCGTGGTATCAGCCTAAAGGTgg                                                                                                                                                                                                                                        >  1:618866/1‑301 (MQ=255)`  
                                                                  `acaggTTCAGGACGTTGTGGTAACTAAAGCGCTCCTTGAGAAGCTACTATCTGACAAACATTACTTCCATCCTGAGATTGACTTTACGGACGTAGGATACACTACGTTCTGGTCAGAATCCCTTGAGGCCGTTGACATTGAACATCGTGCTGCATGGCTGCTCGCTAAACAAGAGCGCAACGGGTTCCCGTTTGACACAAAAGCAATCGAAGAGTTGTACGTAGAGTTAGCTTCTCGCCGCGCTGAGTTGCTCCGTAAATTGACCGAAACGTTCGGCTCGTGGTATCAGCCTAAAGGTGGc                                                                                                                                                                                                                                       <  1:633463/297‑1 (MQ=255)`  
                                                                      `gTTCAGGACGTTGTGGTAACTAAAGCTCTCCTTGAGAAGCTACTCTCTGACAAACATTACTTCCCTCCTGAGATTGACTTTACGGACGTAGGATACACTACGTTCTGGTCAGAATCCCTTGAGGCCGTTGACATTGAACATCGTGCTGCATGGCTGCTCGCTAAACAAGAGCGCAACGGGTTCCCGTTTGACACAAAAGCAATCGAAGAGTTGTAAGTAGAGTTAGCTTCTCGCCGCTCTGAGTTGCTCCGTAAATTGACCGAAACGTTCGGCTCGTGGTATCAGCCTAAAGGTGGCACTg                                                                                                                                                                                                                                   >  1:124479/1‑301 (MQ=255)`  
                                                                         `cAGGCCGTTGTGGTAACTAAAGCTCTCCTTGGTAAGCTACTCTCTGACAAACATTACTTCCCTCCTGAGATTGACTTGACGGACGTAGGATACACTACGTTCTGGTCAGAAGCCCTTGAGTCCGTTGACATTGAACATCGTGCTGCATGGCTGCTCGCTAAACAAGAGCGCAACGGGTTCCCGTTTGACACAAAAGCAATCGAAGAGTTGTACGTAGAGTTAGCTTCTCGCCGCTCTGAGTTGCTCCGTAAATTGACCGAAACGTTCGGCTCGTGGTATCAGCCTAAAGGTGGCACTgaga                                                                                                                                                                                                                                <  1:182758/301‑1 (MQ=255)`  
                                                                         `cAGGACGTTGTGGTAACTAAAGCTCTCCTTGAGAAGCTACTCTCTGACAAACATTACTTCCCTCCTGAGATTGACTTTACGGACGTAGGATACACTACGTTCTGGTCAGAATCCCTTGAGGCCGTTGACATTGAACATCGTGCTGCATGGCTGCTCGCTAAACAAGAGCGCAACGGGTTCCCGTTTGACACAAAAGCAATCGAAGAGTTGTACGTAGAGTTAGCTTCTCGCCGCTCTGAGTTGCTCCATAAATTGACCGAAACGTTCGGCTCGTGGTATCAGCCTAAAGGTGGCACTgaga                                                                                                                                                                                                                                <  1:593238/301‑1 (MQ=255)`  
                                                                            `gACGTTGTGGTAACTAAAGCTCTCCTTGAGAAGCTACTCTCTGACAAACATTACTTCCCTCCTGAGATTGACTTTACGGACGTAGGATACACTACGTTCTGGTCAGAATCCCTTGAGGCCGTTGACATTGAACATCGTGCTGCATGGCTGCTCGCTAAACAAGAGCGCAACGGGTTCCCGTTTGACACAAAAGCAATCGAAGAGTTGTACGTAGAGTTAGCTTCTCGCCGCTCTGAGTTGCTCCGTAAATTGACCGAAACGTTCGGCTCGTGGTATCAGCCTAAAGGTGGCACTGAGATGt                                                                                                                                                                                                                             <  1:2783/301‑1 (MQ=255)`  
                                                                              `ggagatgtgtataagagacagccTTGAGAAGCTACTCTCTGACAAACATTACTTCCCTCCTGAGATTGACTTTCCGGACGTAGGATACACTACGTTCTGGTCAGAATCCCTTGAGGCCGTTGACATTGAACATCGTGCTGCATGGCTGCTCGCTAAACAAGAGCGCAACGGGTTCCCGTTTGACACAAAAGCAATCGAAGAGTTGTACGTAGAGTTAGCTTCTCGCCGCTCTGAGTTGCTCCGTAAATTGACCGAAACGTTCGGCTCGTGGTATCAGCCTAAAGGTGGCACTGAGATGTTc                                                                                                                                                                                                                           <  1:387189/280‑1 (MQ=255)`  
                                                                              `cGTTGTGGTAACTAAAGCGCTCCTTGAGAAGCTACTCTCTGACAAACATTACTTCCCTCCTGTGATTGACTTTACGGACGTAGGATACACTACGTTCTGGTCAGAATCCCTTGAGGCCGTTGACATTGAACATCGTGCTGCATGGCTGCTCGCTAAACAAGAGCGCAACGGGTTCCCGTTTGACACAAAAGCAATCGAAGAGTTGTACGTAGAGTTAGCTTCTCGCCGCTCTGAGTTGCTCCGTAAATTGACCGAAACGTTCGGCTCGTGGTATCAGCCTAAAGGTGGCACTGAGATGTTc                                                                                                                                                                                                                           <  1:557015/301‑1 (MQ=255)`  
                                                                              `cGTTGTGATAACTAAAGCTCTCCTTGGGAAGCTACTCTCTGACAAACATTACTTCCCTCCTGAGATTGACTTTACGGACGTAGGATACACTACGTTCTGGTCAGAATCCCTTGAGGCCGTTGAACTTGGACATCGGGCTGCATGGCTGCTCGCTAAACAAGAGCGCAACGGGTTCCCGTTTGACACAAAAGCAATCGAAGAGTTGTACGTAGAGTTAGCTTCTCGCCGCTCTGAGTTGCTCCGTAAATTGACCGAAACGTTCGGCTCGTGGTATCAGCCTAAAGGTGGCACTGAGATGTTc                                                                                                                                                                                                                           <  1:659225/301‑1 (MQ=255)`  
                                                                                    `ggTAACTAAAGCTCTCCTTGAGAAGCTACTCTCTGACAAACATTACTTCCCTCCTGAGATTGACTTTACGGACGTAGGATACACTACGTTCTGGTCAGAATCCCTTGAGGCCGTTGACACTGAACATCGTGCTGCATAGCTGCTAGCTAAACAAGAGCGCAACGGGTTCCCGTTTGACACAAAAGCAATCGAAGAGTTGTACGTAGAGTTAGCTTCTCGCCGCTCTGAGTTGCTCCGTAAATTGACCGAAACGTTCGGCTCGTGGTATCCGccctgtctcttatacacctctccgagcccc                                                                                                                                                                                                                     >  1:299506/1‑273 (MQ=255)`  
                                                                                     `acagcTAAAGCTCTCCTTGAGAAGCTACTCTCTGACAAACATTACTTCCCTCCTGAGATTGACTTTACGGACGTAGGATACACTACGTTCTGGTCAGAATCCCTTGAGGCCGTTGACATTGAACATCGTGCTGCATGGCTGCTCGCTAAACAAGAGCGCAACGGGTTCCCGTTTGACACAAAAGCAATCGAAGAGCTGTACGTAGAGTTAGCTTCTCGCCGCTCTGAGTTGCTCCGTAAATTGACCGAAACGTTCGGCTCGTGGTATCAGCCTAAAGGTGGCACTGAGATGTTCTGCCATc                                                                                                                                                                                                                    <  1:100034/297‑1 (MQ=255)`  
                                                                                       `aaCTAAAGCTCTCCTTGAGAAGCTACTCTCTGACAAACATTACTTCCCTCCTGAGATTGACTTTACGGACGTAGGATACACTACGTTCTGGTCAGAATCCATTGAGGCCGTTGACATTGAACATCGTGCTGCATGGCTGCTCGCTAAACAAGAGCGCAACGGGTTCCCGTTTGACACAAAAGCAATCGAAGAGTTGTACGTAGAGTTAGCTTCTCGCCGCTCTGAGTTGCTCCGTAAATTGACCGAAACGTTCGGCTCGTGGTATCAGCCTAAAGGTGGCACTGAGATGTTCTGCCATcac                                                                                                                                                                                                                  >  1:328573/1‑299 (MQ=255)`  
                                                                                         `cTAAAGCTCTCCTTGAGAAGCTACTCTCTGACAAACATTACTTCCCTCCTGAGATTGACTTTACGGACGTAGGATACACTACGTTCTGGTCAGAATCCCTTGAGGCCGTTGACATTGAACCTCGTGCTGCATGGCTGCTCGCTAAACAAGAGCGCAACGGGTTCCCGTTTGGCACAAAAGCAATCGAAGAGTTGTACGTAGAGTTAGCTTCTCGCCGCTCTGCGTTGCTCCGTAAATTGACCGAAACGTTCGGCCCGTGGTATCAGCCTAACGGTGGCACTGAGATGTTCTGCCATCcgcg                                                                                                                                                                                                                >  1:222471/1‑301 (MQ=255)`  
                                                                                               `ataagaaacagaAGCTACTCTCTGACAAACATTACTTCCCGCCTTAGATTAGCTTTACGGACGTAGTATACACTACGTTCTTGTCAGAATCCCTGGAGGCCGTTGACATTGAACAGCGTGCTGCATGGCTGCTCGCGAAACAAGCGCGCAACGGGTTCCCGTTTGACGCAAAAGCAATCGAAGAGTTGTACGTAGAGTTAGCTTCTCGCCGCTCTGAGTTGCTCCGTAGATTGACCGAGACGTTCGGCTCGTGGTATCAGCCTAAAGGTGGCACTGAGATGTTCTGCCATCCGCGAACAgg                                                                                                                                                                                                          <  1:248306/292‑1 (MQ=255)`  
                                                                                                 `ctcCTTGAGAAGCTACTCTCTGACAAACATTACTTCCCTCCTGCGATTGACTTTACGGACGTAGGATACACTACGTTCTGGTCAGAATCCCTTTAGGCCGTTGACATTGAACATCGTGCTGCATGGATGCTCGCTAAACAAGAGCGCAACGGGTTCCCGTTTGACACAAAAGCAATCGAAGAGTTGTACGTAGAGTTAGCTTCTCGCCGCTCTGAGTTGCTCCGTAAATTGACCGAAACGTTCGGCTCGTGGTATCAGCCTCAAGGTGGCACTGAGATGTTCTGCCATCCGCGAACAGGTa                                                                                                                                                                                                        >  1:216777/1‑301 (MQ=255)`  
                                                                                                 `ctcCTTGAGAAGCTACTCTCTGACAAACATTACTTCCCTCCTGAGATTGACTTTACGGACGTAGGATACACTACGTTCTGGTCAGAATCCCTTGAGGCCGTTGACATTGAACATCGTGCTGCATGGCTGCTCGCTAAACAAGAGCGCAAAGGGTTCCCGTTTGACACAAAAGCAATCGAAGAGTTGTACGTAGAGTTAGCTTCTGTCCGATCTGAGTTGATCCGTAAATTGACCGAAACGTTCGGCTCGTTGTATCAGACTAAAGGTGTCACTGAGAGGTTCTGCCATCCGAGCACAGTTa                                                                                                                                                                                                        >  1:451349/1‑301 (MQ=255)`  
                                                                                                 `ctcCTTGAAAAGCTACTCTCTGACAAACATTACTTCCCTCCTGAGATTGACTTTACGGACGTAGGATACACTACGTTCTGGTCAGAATCCCTTGAGGCCGTTGACATTGAACATCGTGCTGCATGGCTGCTCGCTAAACAAGAGAGCAACGGGTTCCCGTTTGACACAAAAGCAATCGAAGAGTTGTACGTAGAGTTAGCTTCTCGCCGCTCTGAGTTGCTCCGTAAATTGACCGACACGTTCGGCCCGTGGTATCAGCCTAAAGGTGGCCCTGAGAGGTTATGCCATACTCGAACAGGTa                                                                                                                                                                                                        >  1:345620/1‑301 (MQ=255)`  
                                                                                                   `ccTTGAGAAGCTACTCTCTGACAAACATTACTTCCCTCCTGAGCTTGACTTTACGGACGTAGGATACACTACGTTCTGGTCAGAATCCCTTGAGGCCGTTGACATTGAACATCGTGCTGCATGGCTGCTCGCTAAACAAGAGCGCAACGGGTTCCCGTGTGACACAACAGCCATCGAAGAGTTGTACGTAGCGTTAGCTTCTCGCCGCTCTGAGTTGCTCCGTAAATTAACCGCACCGTTCGGCCCGTGGTATCACCCTCAAGGTAGCACTGCGATCTTCTGCCATCCTCGCACAGGTaac                                                                                                                                                                                                      >  1:485785/1‑300 (MQ=255)`  
                                                                                                     `ttGAGAAGCTACTCTCTGACAAACATTACTTCCCTCCTGAGATTGACTTTACGGACGTAGGATACACTACGTTCTGGTCAGAATCCCTTGAGGCCGTTGACATTGAACATCGTGCTGCATGGCTGCTCGCTAAACAAGAGCGCAACGGGTTCCCGTTTGACACAAAAGCAATCGAAGAGTTGTACGTAGAGTTAGCTTCTCGCCGCTCTGAGTTGCTCCGTAAATTGACCGAAACGTTCGGCTCGTGGTATCAGCCTAAAGGTGGCACTGAGATGTTCTGCCATCCGCGAACAGGTAAGcc                                                                                                                                                                                                    <  1:267112/301‑1 (MQ=255)`  
                                                                                                       `gagaAACTACTATTTGACAAACATTACTGCCCTCCTGAGAGTGACTTTACGGACGTAGGATACACTACGTTCTGGTCAGAATCCCTTGAGGCCGTTGACATTGAACATCGTGCTGCATGGCTGCTCGCTAAACAAGAGCGCAACGGGTTCCCGTTTGACACAAAAGCAATCGAAGAGTTGTACGTAGAGTTAGCTTCTCGCCGCTCTGAGTTGCTCCGTAAATTGACCGAAACGTTCGGCTCGTGGTATCAGCCTAAAGGTGGCACTGAGATGTTCTGCCATCCGCGAACAGGTAAGCCAc                                                                                                                                                                                                  <  1:475323/301‑1 (MQ=255)`  
                                                                                                               `aCTCTCTGACAAACATTACTTCCCTCCTGAGATTGACTTTACGGACGTAGGATACACTACGTTCTGGTCAGAATCCCTTGAGGCCGTTGACATTGAACATCGTGCTGCATGGCTGCTCGCTAAACAAGAGCGCAACGGGTTCCCGTTTGACACAAAAGCAATCGAAGAGTTGTACGTAGAGTTAGCTTCTCGCCGATCTGAGTTGCTCCGTAAATTGACCGAAACGTTCGGCTCGTGGTATCAGCCTAAAGGTGGCACTGAGCTGTTCTGCCATCCTCGAACAGGTAAGCCACTCCCTtaa                                                                                                                                                                                          >  1:381641/1‑301 (MQ=255)`  
                                                                                                                `ctctctGACAAACATTACTTCCCTCCTGAGATTGACTTTACGGACGTAGGATACACTACGTTCTGGTCAGAATCCCTTGAGGCCGTTGACATTGAACATCGTGCTGCATGGCTGCTCGCTAAACAAGAGCGCAACGGGTTCCCGTTTGACACAAAAGCAATCGAAGAGTTGTACGTAGAGTTAGCTTCTCGCCGCTCTGAGTTGCTCCGTAAATTGACCGAAACGTTCGGCTCGTGGTATCAGCCTAAAGGTGGCACTGAGATGTTCTGCCATCCGCGAACAGGTAAGCCACTACCTAAAt                                                                                                                                                                                         <  1:241577/301‑1 (MQ=255)`  
                                                                                                                   `tctGACAAACATTACTTCCCTCCTGAGATTGACTTTACGGACGTAGGATACACTACGTTCTGGTCAGAATCCCTTGAGGCCGTTGACATTGAACATCGTGCTGCATGGCTGCTCGCTAAACAAGAGCGCAACGGGTTCCCGTTTGACACAAAAGCAATCGAAGAGTTGTACGTAGAGTTAGCTTCTCGCCGCTCTGAGTTGCTCCGTAAATTGACCGAAACGTTCGGCTCGTGGTATCAGCCTAAAGGTGGCACTGAGATGTTCTGCCATCCGCGAACAGGTAAGCCACTACCTAAATAcc                                                                                                                                                                                      <  1:272081/301‑1 (MQ=255)`  
                                                                                                                   `caggACTAACATTCCTGCCCTCCTGAGATGTACTTTACGGACGTAGGATACACTACGTTCTGGTCAGAATCCCTTGAGGCCGTTGACACTGAACATCGTGCTGCATGGCTGCTCGCTAAACAAGAGCGCAACGGGTTCCCGTTTGACACAAAAGCAATCGAAGAGTTGTACGTAGAGTTAGCTTCTCTCCGCTCTGAGTTGCTCCGTAAATTGACCGAAACATTCGGCTCGTGGTATCAGCCTAAAGGTGGCACTGATATGTTCTGCCATCCGCGAACAGGTAAGCCACTACCTAAATAcc                                                                                                                                                                                      <  1:88286/298‑1 (MQ=255)`  
                                                                                                                      `gACAAACATTACTTCCCTCCTGAGATTGACTTTACGGACGTAGGATACACTACGTTCTGGTCAGAATCCCTTGAGGCCGTTGACATTGAACATCGTGCTGCATGGCTGCTCGCTAAACAAGAGCGCAACGGGTTCCCGTTTGACACAAAAGCAATCGAAGAGTTGTACGTAGAGTTAGCTTCTCGCCGCTCTGAGTTGCTCCGTAAATTGACAGAAACGTTCGGCTCGTGGTATCAGCCTAAAGGTGGCACTGCGATGTTCTGCCATCCGCGAACAGGTAAGCCACTACCTAAATACCCTc                                                                                                                                                                                   >  1:585932/1‑301 (MQ=255)`  
                                                                                                                      `gACAAACATTACTTCCCTCCTGAGATTGACTTTACGGACGTAGGATACACTACGTTCTGGTCAGAATCCCTTGAGGCCGTTGACATTGAACATCGTGCTGCATGGCTGCTCGCTAAACAAGAGCGCAACGGGTTCCCGTTTGAAACAAAACCAATCGAAGAGTTGTACGTAGCGTTAGCTTCTCGCCGCCCTGAGTTGCTCCGTAACTTGAACGAAACGGTCGTCCCGTGGTATAAGCCTAAAGGTGGCCCTGAACTGTTCAGCTATCCGCGACCAGGTAACCCACTACCTACATACCCTc                                                                                                                                                                                   >  1:580399/1‑301 (MQ=255)`  
                                                                                                                      `gACAAACATTACTTCCCTCCTGAGATTGACTTTACGGACGTAGGATACACTACGTTCTGGTCAGAATCCATTGAGGCCGTTGACATTGAACATCGTGCTGCATGGCTGCTCGCTAAACAAGAGCGCAACGGGTTCCCGTTTGACACAAAAGCAATCGAAGAGTTGTACGTAGAGTTAGCTTCTCGCCGCTCTGAGTTGCTCCGTAAATTGACCGAAACGTTCGGCTCGTGGTATCAGCCTAAAGGTGGCACTGAGTTGTTCTGCCATCCGCGAACAGGTAAGCCACTACCTAAATActgtc                                                                                                                                                                                   >  1:459190/1‑297 (MQ=255)`  
                                                                                                                            `cATTACTTCCCTCCTGAGATTGACTTTACGGACGTAGGATACACTACGTTCTGGTCAGAATCCCTTGAGGCCGTTGACATTGAACATCGTGCTGCATGGCTGCTCGCTAAACAAGAGCGCAACGGGTTCCCGTTTGACACAAAAGCAATCGAAGAGTTGTACGTAGAGTTAGCTTCTCGCCGCTCTGAGTTGCTCCGTAAATTGACCGAAACGTTCGGCTCGTGGTATCAGCCTAAAGGTGGCACTGAGATGTTCTGCCATCCGCGAACAGGTAAGCCACTACCTAAATACCCTCGCAttt                                                                                                                                                                             >  1:76272/1‑300 (MQ=255)`  
                                                                                                                            `cATTACTTCCCTCCTGAGATTGACTTTACGGACGTAGGATACACTACGTTCTGGTCAGAATCCCTTGAGGCCGTTGACATTGAACATCGTGCTGCATGGCTGCTCGCTAAACAAGAGCGCAACGGGTTCCCGTTTGACACAAAAGCAATCGAAGAGTTGTACGTAGAGTTAGCTTCTCGCCGCTCTGAGTTGCTCCGTAAATTGACCGAAACGTTCGGCTCGTGGTATCAGCCTAAAGGTGGCACTGAGATGTTCTGCCATCCGCGAACAGGTAAGCCACTACCTAAATACCCTCGCATTa                                                                                                                                                                             >  1:5375/1‑301 (MQ=255)`  
                                                                                                                             `aTTACTTCCCTCCTGAGATTGATTTTACGGACGTAGGATACACTACGTTCTGGTCAGAATCCCTTGAGGCCGTTGACATTGAACATCGTGCTGCATGGCTGCTCGCTAAACAAGAGCGCAACGGGTTCCCGTTTGACACAAAAGCAATCGAAGAGTTGTACGTAGAGTTAGCTTCTCGCCGCTCTGAGTTGCTCCGT‑AATTGACCGAAACGTTCGGCTCGTGGTATCAGCCTAAAGGTGGCACTGAGATGTTCTGCCATCCGCGAACAGGTAAGCCACTACCTAAATACCCTCGCATTAAg                                                                                                                                                                           <  1:310101/301‑1 (MQ=255)`  
                                                                                                                                `ataagagacaggagaTTGACTTTACGGACGTAGGATACACTACGTTCTGGTCAGAATCCCTTGAGGCCGTTGACATTGAACATCGTGCTGCATGGCTGCTCGCTAAACAAGAGCGCAACGGGTTCCCGTTTGACACAAAAGCAATCGAAGAGTTGTACGTAGAGTTAGCTTCTCGCCGCTCTGAGTTGCTCCGTAAATTGACCGAAACGTTCGGCTCGTGGTATCAGCCTAAAGGTGGCACTGAGATGTTCTGCTATCCGCGAACAGGTAAGCCACTACCTAAATACCCTCGCATTAAGac                                                                                                                                                                         <  1:464282/290‑1 (MQ=255)`  
                                                                                                                                   `tCCCTCCTGAGATTGACTTTACGGACGTAGGATACACTACGTTCTGGTCAGAATCCCTTGAGGCCGTTGACATTGAACATCGTGCTGCATGGCTGCTCGCTAAACAAGAGCGCAACGGGTTCCCGTTTGACACAAAAGCAATCGAAGAGTTGTACGTAGAGTTAGCTTCTCGCCGCTCTGAGTTGCTCCGTAAATTGACCGAAACGTTCGGCTCGTGGTATCAGCCTAAAGGTGGCACTGAGATGTTCTGCCATCCGCGAACAGGTAAGCCACTACCTAAATACCCTCGCATTAAGacctt                                                                                                                                                                      >  1:416983/1‑298 (MQ=255)`  
                                                                                                                                   `tCCCTCCTGAGATTGACTTTACGGACGTAGGATACACTACGTTCTGGTCAGAATCCCTTGAGGCCGTTGACATTGAACATCGTGCTGCATGGCTGCTCGCTAAACAAGAGCGCAACGGGTTCCCGTTTGACACAAAAGCAATCGAAGAGTTGTACGTAGAGTTAGCTTCTCGCCGCTCTGAGTTGCTCCGTAAATTGACCGAAACGTTCGGCTCGTGGTATCAGCCTAAAGGTGGCACTGAGATGTTCTGCCATCCGCGAACAGGTAAGCCACTACCTAAATACCCTCGCATTAAGACAcc                                                                                                                                                                      >  1:93521/1‑301 (MQ=255)`  
                                                                                                                                        `cctGAGATTGACTTTACGGACGTAGGATACACTCCGTTCTGTTCAGCATCCCTTGAGGCCGTTGACATTGAACATCGTGCTGCATGTCTGCGCGCTAAACAAGAGCGCAACGGGTTCCCGTTTGACACAAAAGCAATCGAAGAGTTGTACGTAGAGTTAGCTTCTCGCCGCTCTGAGTTGCTCCGTAAATTGACCGAAACGTTCGGCTCGTGGTATCAGCCTAAAGGTGGCACTGAGATGTTCTGCCATCCGCGAACAGGTAAGCCACTACCTAAATACCCTCGCATTAAGACACCTAAAg                                                                                                                                                                 <  1:520364/301‑1 (MQ=255)`  
                                                                                                                                        `cctGAGATTGACTTTACGGACGTAGGATACACTACGTTCTGGTCAGAATCCCTTGAGGCCGTTGACATTGAACATCGTGCTGCATGGCTGCTCGCTAAACAAGAGCGCAACGGGTTCCCGTTTGACACAAAAGCAATCGAAGAGTTGTACGTAGAGTTAGCTTCTCGCCGCTCTGAGTTGCTCCGTAAATTGACCGAAACGTTCGGCTCGTGGTATCAGCCTAAAGGTGGCACTGAGATGTTCTGCCATCCGCGAACAGGTAAGCCACTACCTAAATACCCTCGCATTAAGACACCTAAAg                                                                                                                                                                 <  1:176207/301‑1 (MQ=255)`  
                                                                                                                                         `ctGAGATTGACTTTACGGACGTAGGATACACTACGTTCTGGTCAGAATCCCTTGAGGCCGTTGACATTGAACATCGTGCTGCATGGCTGCTCGCTAAACAAGAGCGCAACGGGTTCCCGTTTGACACAAAAGCAATCGAAGAGTTGTACGTAGAGTTAGCTTCTCGCCGCTCTGAGTTGCTCCGTAAATTGACCGAAACGTTCGGCTCGTGGTATCAGCCTAAAGGTGGCACTGAGATGTTCTGCCATCCGCGAACAGGTAAGCCACTACCTAAATACCCTCGCATTAAGACACCTAAAGt                                                                                                                                                                <  1:82003/301‑1 (MQ=255)`  
                                                                                                                                               `ttGACTTTACGGACGTAGGATACACTACGTTCTGGTCAGAATCCCTTGAGGCCGTTGACATTGAACATCGTGCTGCATGGCTGCTCGCTAAACAAGAGCGCAACGGGTTCCCGTTTGACACAAAAGCAATCGAAGAGTTGTACGTAGAGTTAGCTTCTCGCCGCTCTGAGTTGCTCCGTAAATTGACCGAAACGTTCGGCTCGTGGTATCAGCCTAAAGGTGGCACTGAGATGTTCTGCCATCCGCGAACAGGTAAGCCACTACCTAAATACCCTCGCATTAAGACACCTAAAGTtggtgg                                                                                                                                                          <  1:43432/301‑1 (MQ=255)`  
                                                                                                                                                 `gACTTTACGGACGTAGGATACACTACGTTCTGGTCAGAATCCCTTGAGGCCGTTGACATTGAACATCGTGCTGAATGGCTGCTCGCGAAACAAGAGCGCAACGGGTTCCCGTTTGACACAAAAGCAATCGAAGAGTGGTACGTAGAGTTAGCTTCTCGCCGCTCTGAGTTGCTACGTAAATTGACCGAACAGTTCGGCTCGTGGTATCAGCCTAAAGGTGGCACTGAGATATTATGCCATCCGCGAACAGGTAAGCCACTACCTAAATACCCTCGCATTAAGACACCTAAAGTTGGTGTTa                                                                                                                                                        >  1:525653/1‑301 (MQ=255)`  
                                                                                                                                                  `aCTTTACGGACGTAGGATACACTACGTTCTGGTCAGAATCCCTTGAGGCCGTTGACATTGAACATCGTGCTGCATGGCTGCTCGCTAAACAAGAGCGCAACGGGTTCCCGTTTGACACAAAAGCAATCGAAGAGTTGTACGTAGAGTTAGCTTCTCGCCGCTCTGAGTTGCTCCGTAAATTGACCGAAACGTTCGGCTCGTGGTATCAGCCTAAAGGTGGCACTGAGATGTTCTGCCATCCGCGAACAGGTAAGCCACTACCTAAATACCCTCGCATTAAGACACCTAAAGTTGGTGGTAt                                                                                                                                                       <  1:515735/301‑1 (MQ=255)`  
                                                                                                                                                   `cTTTACGGACGTAGGATACACTACGTTCTGGTCAGAATCCCTTGAGGCCGTTGACATTGAACATCGTGCTGCATGGCTGCTCGCTAAACAAGAGCGCAACGGGTTCCCGTTTGACACAAAAGCAATCGAAGAGTTGTACGTAGAGTTAGCTTCTCGCCGCTCTGAGTTGCTCCGTAAATTGACCGAAACGTTCGGCTCGTGGTATCAGCCTAAAGGTGGCACTGAGATGTTCTGCCATCCGCGAACAGGTAAGCCACTACCTAAATACCCTCGCATTAAGACACCTAAAGTTGGTGGTATc                                                                                                                                                      <  1:334194/301‑1 (MQ=255)`  
                                                                                                                                                          `gACGTAGGATAAACTACGTTCTGGTCAGAATCCCTTGAGGCCGTTGACATTGAACATCGTGCTGCATGGCTGCTCGCTAAACAAGAGCGCAACGGGTTCCCGTTTGACACAAAAGCAATCGAAGAGTTGTACGTAGAGTTAGCTTCTCGCCGCTCTGAGTTGCGCCGTAAATTGACCGAAACGTTCGGCTCGTGGTATCAGCCTAAAGGTGGCACTGAGATGTTCTGCCATCCGCGAACAGGTAAGCCACTACCTAAATACCCTCGCATTAAGACACCTAAAGTTGGTGGTATCTTTaaga                                                                                                                                               <  1:284966/301‑1 (MQ=255)`  
                                                                                                                                                                `ggATACACTACGTTCTGGTCAGAATCCCTTGAGGCCGTTGACATTGAACATCGTGCTGCATGGCTGCTCGCTAAACAAGAGCGCAACGGGTTCCCGTTTGACACAAAAGCAATCGAAGAGTTGTACGTAGAGTTAGCTTCTCGCCGCTCTGAGTTGCTCCGGAAATTGACCGAAACGTTCGGCTCGTGGTATCAGCCTAAAGGTGGCACTGAGATGTTCTGCCATCCGCGAACAGGTAAGCCACTACCTAAATACCCTAGCATTAAGGAAACTAAAGGTGGTGGTATCTTGAAGAAGCCTa                                                                                                                                         >  1:49230/1‑301 (MQ=255)`  
                                                                                                                                                                    `acacTACGTTCGGGTCAGAATCCCTTGAGTCCGTTGACATTGAACATCGTGCTGCATGGCTGCTCGCTAAAAAAGAGCGCAACGGGTTCCCGTTTGACACAAAAGCAATCGAAGAGTTGTACGTAGAGTTAGCTTCTCGCCGCTCTGAGTTGCTCCGTAAATTGACCGAAACGTTCGGCTCGTGGTATCAGCCTAAAGGTGGCACTGAGATGTTCTGCCATCCGCGAACAGGTAAGCCACTACCTAAATACCCTCGCATTAAGACACCTAAAGTTGGTGGTATCTTTAAGAAGCCTAAGaa                                                                                                                                     <  1:54457/301‑1 (MQ=255)`  
                                                                                                                                                                     `cacTACGTTCTGGTCAGAATCCCTTGAGGCCGTTGACATTGAACATCGTGCTGCATGGCTGATCGCTAAACAAGAGCGCAACGGGTTCCCGTTTGACACAAAAGCAATCGAAGAGTTGTACGTAGAGTTAGCTTCTCGCCGCTCTGAGTTGCTCCGTAAATTGACCGAAACGTTCGGCTCGTGGTATCAGCCTAAAGGTGGCACTGAGATGTTCTGCCATCCGCGAACAGGTAAGCCACTAACTAAATACCCTCGCATTAAGACACCTCAAGTTGGTGGTATCTTTAAGAAGCCTAATAAc                                                                                                                                    >  1:392391/1‑301 (MQ=255)`  
                                                                                                                                                                      `acTACGTTCTGGTCAGAATCCCTTGAGGCCGTTGACATTGAACATCGTGCTGCATGGCTGCTCGCTAAACAAGAGCGCAACGGGTTCCCGTTTGACACAAAAGCAATCGTAGAGTTGTACGTAGAGTTAGCTTCTCGCCGCTCTGAGTTGCTCCGTAAATTGACCGAAACGTTCGGCTCGTGGTATCAGCCTAAAGGTGGCACTGAGATGTTCTGCCATCCGCGAACAGGTAAGCCACTACCTAAATACCCTCGCATTAAGACACCTAAAGTTGGTGGTATCTTTaagactgtctcttata                                                                                                                                   >  1:509042/1‑289 (MQ=255)`  
                                                                                                                                                                         `aCGTTCTGGTCAGAATCCCTTGAGGCCGTTGACATTGAACATCGTGCTGCATGGATGCTCGCTAAACAAGAGCGCAACGGGTTCCCGTTTGAAACAAAAGCTATCGAAGAGTTATACGTAGAGTTAGCTTCTCCCCGCACGGAGTTGCACAGTAAATTGCCCGCAACGTTCGTCTCCTGATCTCAGCCTACAGGTGGCACTGTACTGTTATTCACTCCGCGAACAGGTAAGCACCTACCTAAATACCCTCGCATTAAGCCACCTAAATTTGGTGGTATATTTTGGAATCCTAAGAGCaaag                                                                                                                                >  1:104858/1‑299 (MQ=255)`  
                                                                                                                                                                          `gtgtagaagagacggcccATGAGGTCGGTGACAGTGATCAGCGTTGTGCATGGCTGCTCGCTAAACAAGATTGCAACGGGTTCCCGGTTGACAAAGAAGCAATCGAAGGGTTGTACGTAGAGTTAGCTTCTCGGCGCGCGGAGTTGCTCCGTAAATTGACCTAAACGTTCGGCTCGGGGTATCAGCCTAAAGGTGGCAATGAGATGGTCTGCCATCCGCGAACAGGTAACCCACTACCGAAATACCCGCGCATTAAGACACCTAAAGTTGGTGGTATCTTTAAGAAGGCTAAGAACAAGGc                                                                                                                               <  1:291709/286‑1 (MQ=255)`  
                                                                                                                                                                          `cGTTCAGGTCAGAATCCCTGGAGGCAGTTGACGTTGAACATCAAGCTGCATGGCTGCTCACAAAACAAGAGAGCAACGGGTTGCCGTTTGAAACAAAAATAATCGAAGAGTTGTACGTAGAGTTAGCTTCTAGCCGCTCTGAGGTGCCCCGAAGATTGACCGAAACGTTCGGCTCGTGGTATAAGCCTAAAGGTGGCACTGAGATGTTCTGCCATCCGCGAACAGGTAAGCCACTACCTAAATACCCTCGCATTAAGACCCCTAAAGTTGGTAGTATCTTTAAGAAGCCTAAGAACAAGGc                                                                                                                               <  1:625514/301‑1 (MQ=255)`  
                                                                                                                                                                           `gTTCTGGTCAGAATCCCTTGAGGCCGTTGACATTGAACATCGTGCTGCATGGCTGCTCGCTAAACAAGAGCGCAACGGGTTCCCGTTTGACACAAAAGCAATCGAAGAGTTGTACGTAGAGTTAGCTTCTCGCCGATCTGAGTTGCTCCGTAAATTGACCGAAACGTTCGGCTCGTGGTATCAGCCTAAAGGTGGCACTGAGATGGTCTGCCATCCGCGAACAGGTAAGCCACTACTTAAATACCCTCGCATTAAGACACCTAAAGTTGGTGGTATCTTTAAGAAGCCTAAGAAACAGGca                                                                                                                              >  1:398438/1‑301 (MQ=255)`  
                                                                                                                                                                                `ggTCAGAATCCCTTGAGGCCGTTGACATTGAACATCGTGCTGCATGGCTGCTCGCTAAACAAGAGCGCAACGGGTTCCCGTTTGACACAAAAGCAATCGAAGAGTTGTACGTAGAGTTAGCTTCTCGCCGCTCTGAGTTGCTCCGTAAATTGACCGAAACGTTCGGCTCGTGGTATCAGCCTAAAGGTGGCACTGAGATGTTCTGCCATCCGCGAACAGGTAAGCCACTACCTAAATACCCTCGCATTAAGACACCTAAAGTTGGTGGTATCTTTAAGAAGCCTAAGAACAAGGCACAGCg                                                                                                                         <  1:428258/301‑1 (MQ=255)`  
                                                                                                                                                                                 `gTCAGAATCCCTTGAGGCCGTTGACATTGAACATCGTGCTGCATGGCTGCTCGCTAAACAAGAGCGCAACGGGTTCCCGTTTGACACAAAAGCAATCGAAGAGTTGTACGTAGAGTTAGCTTCTCGCCGCTCTGAGTTGCTCCGGAAATTGACCGAAACGTTCGGCTCGTGGTATCAGCCTAAAGGTGGCACTGAGATGTTCTGCCATCCGCGAACAGGTAAGCCACGACCGAAATACACTCGCATTAAGACACGTAAAGTTGGTGGTATCGTGAAGAAGCCTAAGAACAAGGCACAGCga                                                                                                                        >  1:315617/1‑301 (MQ=255)`  
                                                                                                                                                                                       `ataccTTAAGGCCGATGACATTGAACATCGTGCTGCATGGCTGCTCGCAAAAGAAGAGCGCAACAGGTTCCCGTTTAAAACAAAAGCAATCTAAGAGTTTTACGGAGAGTGAGCTTCACGCCGCTCTGTGATGCTCCGTAAATTGACCGAAACGTTCGGCACGTGGTATCAGCCTAAAGGTGGCACTGAGATGTTCTGCCATCCGCGAAAAGGTAAGTCACTACCTAAATACCCTCGCATTAAGACACCTAAAGTTGGTGGTATCTTTAAGAAGCCTAAGAACAAGACACAGCGAGAAGGc                                                                                                                  <  1:137568/298‑1 (MQ=255)`  
                                                                                                                                                                                                 `gCCGTTGACATTGAACATCGTGCTGCATGGCTGCTCGCTAAACAAGAGCGCAACGGGTTCCCGTTTGACACAAAAGCAATCGAAGAGTTGTACGTAGAGTTAGCTTCTCGCCGCTCTGAGTTGCTCCGTAAATTGACCGAAACGTTCGGCTCGTGGTATCAGCCTAAAGGTGGCACTGAGATGTTCTGCCATCCGCGAACAGGTAAGCCACTACCTACATACCCTCGCATTAAGACACCTAAAGTTGGTGGTATCTTTAAGAAGCCTAAGAACAAGGCACAGCGAGAAGGCCGTGAGccct                                                                                                        >  1:607583/1‑299 (MQ=255)`  
                                                                                                                                                                                                     `tcggagatgtgtataagagacagtGGCTGCTCGCTAAACAAGAGCGCAACGGGTTCCCGTTTGACACAAAAGCAATCGAAGAGTTGTACGTAGAGTTAGCTTCTCGCCGCTCTGAGTTGCTCCGTAAATTGACCGAAACGTTCGGCTCGTGGTATCAGCCTAAAGGTGGCACTGAGATGTTCTGCCATCCGCGAACAGGTAAGCCACTACCTAAATACCCTCGCATTAAGACACCTAAAGTTGGTGGTATCTTTAAGAAGCCTAATAACAAGGCACAGCGAGAAGGCCGTGAGCCTTGCGa                                                                                                    <  1:151221/278‑1 (MQ=255)`  
                                                                                                                                                                                                                    `gTGCTGCATGGCTGCTCGCTAAACAAGAGCGCAACGGGTTCCCGTTTGACACAAAAGCAATCGAAGAGTTGTACGTAGAGTTAGCTTCTTGCCGCTCTGAGTTGCTCCGTAAATTGCCTGAAATGTTCGGCTTGTGGTATCAGCCTAAAGGGGGCACTGAGATGTTCTGCCATCCGCGAACTGGCAAGCCACTACCTAAATACCTCCCCATTAAGACACCTAAAGTTGGTGGTATCTTTAAGAAGCCTAATAACAAGGCACAGAGAGAAGGCCGTGTGCCTCGCGAACTTGATACCcgcct                                                                                     >  1:228418/1‑299 (MQ=255)`  
                                                                                                                                                                                                                              `gctgctCGCTAAACAAGAGCGCAACGGGTTCCAGTTTGACACAAAAGCAATCGAAGAGTTGTACGTAGAGTTAGCTTCTCGCCGCTCTGAGTTGCTCCGTAAATTGACCGAAACGTTCGGCTCGTGGTATCAGCCTAAAGGTGGCACTGAGATGTTCTGCCATCCGCGAACAGGTAAGCAACTACCTAAATACCCTCGCAATAAGACACATAAAGTTGGTGGTATCTTTAAGAAGCCTAAGACCAAGGCACAGCGAGAAGGCCGTAAGCCTTGCCAACTCGATACCAGAGCGTACGGTGCt                                                                           >  1:445537/1‑301 (MQ=255)`  
                                                                                                                                                                                                                                `tgctCGCTAAAAAAGAGCGCAACGGGTTCCCGGTTGAAACAAAAGCAATCGAAGAGTTGTACGTAGAGTTAGCTTCTCGCCACTCTGAGTTGCTCCGTAAATTGACCGAAACGTTCGGCTCGTGGTATCAGCCTAAAGGTGGCACTGAGATGTTCTGCCATCCGCGAACAGGTAAGCCACTACCTAAATACCCTCGCATGAAGACACCTAAAGTTGGTGGTATCTTTAAGAAGCCTAAGAACAAGGCACAGCGAGAAGGCCGTGAGCCTTGCGAACTTGATACCCGCGAGTACGTTGCTgg                                                                         <  1:422112/301‑1 (MQ=255)`  
                                                                                                                                                                                                                                        `aaaCAAGAGCGCAACGGGTTCCCGTTTGACACAAAAGCAATCGAAGAGTTGTACGTAGAGTTAGCTTCTCGCCGCTCTGAGTTGCTCCGTAAATTGACCGAAACGTTCGGCTCGTGGTATCAGCCTAAAGGTGGCACTGAGATGTTCTGCCATCCGCGAACAGGTAAGCCACTACCTAAAAACCCTCGCATTAAGACACCTAAAGTTGGTGCTATCTTTAAGAAGCCTAAGAACAAGGCACAGCGAGAAGGCCGTGAGCCTTGCGAACTTGATACCCGCGAGTACGTTGCTGGTGCTCCtt                                                                 <  1:11613/301‑1 (MQ=255)`  
                                                                                                                                                                                                                                           `cAAGAGCGCAACGGGTTCCCGTTTGACACAAAAGCAATCGAAGAGTTGTACGTAGAGTTAGCTTCTCGCCGCTCTGAGTTGCTCCGTAAATTGACCGAAACGTTCGGCTCGTGGTATCAGCCTAAAGGTGGCACTGAGATGTTCTGCCATCCGCGAACAGGTAAGCCACTACCTAAATACCCTCGCATTAAGACACCTAAAGTTGGTGGTATCTTTAAGAAGCCTAAGAACAAGGCACAGCGAGAAGGCCGTGAGCCTTGCGAACTTGATACCCGCGAGTACGTTGCTGGTGCTCCTTaca                                                              >  1:37500/1‑301 (MQ=255)`  
                                                                                                                                                                                                                                             `agagCGCAACGTTTTCCCGTTTGACACAAAAGCAATCGAAGAGTTGTACGTAGGTTTAGCTTCTCGCCGCTCTGAGTTGCTCCGTAAATTGACCGAAACGTTCGGCTCGTGGTATCAGCCTAAAGGTGGCACTGAGATGTTCTGCCATCCGCGAACAGGTAAGCCACTACCTAAATACCCTCGCATTAAGACACCTAAAGTTGGTGGTATCTTTAAGAAGCCTAAGAACAAGGCACAGCGAGAAGGCCGTGAGCCTTGCGAACTTGATACCCGCGAGTACGTTGCTGGTGCTCCTTACAcc                                                            <  1:254400/301‑1 (MQ=255)`  
                                                                                                                                                                                                                                              `gagCGCAACGGGTTCCCGTTTGACACAAAAGCAATCGAAGAGTTGTACGTAGAGTTAGCTTCTCGCCGCTCTGAGTTGCTCCGTAAATTGACCGAAACGTTCGGCTCGTGGTATCAGCCTAAAGGTGGCACTGAGATGTTCTGCCATCCGCGAACAGGTAAGCCACTACCTAAATACCCTCGCATTAAGACACCTAAAGTTGGTGGTATCTTTAAGAAGCCTAAGACCAAGGCACAGCGCGAAGGCCGTGAGCATTGCGAACTTGATACCCGCGAGTACGTTGCTGGTGCTCCTTACAccc                                                           >  1:647088/1‑301 (MQ=255)`  
                                                                                                                                                                                                                                                       `gggTTCCCGTTTGACACAAAAGCAATCGAAGAGTTGTACGTAGAGTTAGCTTCTCGCCGCTCTGAGTTGCTCCGTAAATTGACCGAAACGTTCGGCTCGTGGTATCAGCCTAAAGGTGGCACTGAGATGTTCTGCCATCCGCGAACAGGTAAGCCACTACCTAAATACCCTCGCATTAAGACACCTAAAGTTGGTGGTATCTTTAAGAAGCCTAAGAACAAGGCACAGCGAGAAGGCCGTGAGCCTTGCGAACTTGATACCCGCGAGTACGTTGCTGGTGCTCCTTACACCCCAGTTGAAc                                                  <  1:574831/301‑1 (MQ=255)`  
                                                                                                                                                                                                                                                             `gagacagACACAAAAGCAATCGAAGAGTTGTACGTAGAGTTAGCTTCTAGCCGCTCTGAATTGCTCCGTAAATTGACCGAAACGTTCGGCTCGTGGTATCAGCCGAAAGGTGGCACTGAGATGTTCTGCCATCCGCGAACAGGTAAGCCACTACCTAAATACCCTCGCATTAAGACACCTAAAGTTGGTGGTATCTTTAAGAAGCCTAAGAACAAGGCACAGCGAGAAGGCCGTGAGCCTTGCGAACTTGATACCCGCGAGTACGTTGCTGGTGCTCCTTACACCCCAGTTGAACAtgttg                                            <  1:199534/295‑1 (MQ=255)`  
                                                                                                                                                                                                                                                                     `aacaAAAGCAATCGACGAGTTGTACGTAGAGTTAGCTTCTCGCCGCTCTGAATTGCTCCGTAAATTGACCGAAACGTTCGGCTCGTGGTATCAGCCTAAAGGTGGCACTGAGATGTTCTGCCATCCGCGAACAGGTAAGCCACTACCTAAATACCCTCGCATTAAGACACCTAAAGTTGGTGGTATCTTTAAGAAGCCTAAGAACAAGGCACAGCGAGAAGGCCGTGAGCCTTGCGAACTTGATACCCGCGAGTACGTTGCTGGTGCTCCTTACACCCCAGTTGAACATGTTGTGTTGAAc                                    <  1:170535/300‑1 (MQ=255)`  
                                                                                                                                                                                                                                                                     `aacaAAAGCAATCGAAGAGTTGTACGTAGAGTTAGCTTCTCGCCGCTCTGAGTTGCTCCGTAAATTGACCGAAACGTTCGGCTCGTGGTATCAGCCTAAAGGTGGCACTGAGATGTTCTGCCATCCGCGAACAGGTAAGCCACTACCTAAATACCCTCGCATTAAGACACCTAAAGTTGGTGGTATCTTTAAGAAGCCTAAGAACAAGGCACAGCGAGAAGGCCGTGAGCCTTGCGAACTTGATACCCGCGAGTACGTTGCTGGTGCTCCTTACACCCCAGTTGAACATGTTGTGTTTAAc                                    <  1:159421/300‑1 (MQ=255)`  
                                                                                                                                                                                                                                                                        `aaaaGCAATCGAAGAGTTGTACGTAGAGTTAGCTTCTCGCCTCTCTGAGTTGCTCCGTAAATTGACCGAAACGTTCGGCTCGTGGTATCAGCCTAAAGGTGGCACTGAGATGTTCTGCCATCCGCGAACAGGTAAGCCACTACCTAAATACCCTCGCATTAAGACACCTAAAGTTGGTGGTATCTTTAAGAAGCCTAAGAACAAGGCACAGCGAGAAGGCCGTGAGCCTTGCGAACTTGATACCCGCGAGTACGTTGCTGGTGCTCCTTACACCCCAGTTGAACATGTTGTGTTTAACCCt                                 <  1:322934/301‑1 (MQ=255)`  
                                                                                                                                                                                                                                                                             `gacaggAAGAGTTGGACGGAGAGTTAGATTCTCGCCGCTCTTAGTTGCTCCGTAAATTGACCGAAACGTTCGGCTCGTGGTATCAGCCTAAAGGTGGCACTGAGATGTTCTGCCATCCGCGAACAGGTAAGCCACTACCTAAATACCCTCGCATTAAGACACCTAAAGTTGGTGGTATCTTTAAGAAGCCTAAGAACAAGGCACAGCGAGAAGGCCGTGAGCCTTGCGAACTTGATACCCGCGAGTACGTTGCTGGTGCTCCTTACACCCCAGTTGAACATGTTGTGTTTAACCCTTCGtc                            <  1:653423/296‑1 (MQ=255)`  
                                                                                                                                                                                                                                                                             `cAATCGAAGAGTTGTACGTAGAGTTAGCTTCTCGCCGCTCTGAGTTGCTCCGTAAATTGACCGAAACGTTCGGCTCGTGGTATCAGCCTAAAGGTGGCACTGAGATGTTCTGCCATCCGCGAACAGGTAAGCCACTACCTAAATACCCTCGCATTAAGACACCTAAAGTTGGTGGTATTTTTAAGAAGCCTAAGAACAAGGCACAGCGAGAAGGCCGTGAGCCTTGCGAACTTGATACCCGCGAGTACGTTGCTGGTGCTCCTTACACCCCAGTTGAACAtgttgctgtctcttatacaca                            >  1:145334/1‑285 (MQ=255)`  
                                                                                                                                                                                                                                                                               `aTCGAAGAGTTGTACGTAGAGTTAGCTTCTCGCCGCTCTGAGTTGCTCCGTAAATTGACCGAAACGTTCGGCTCGTGGTATCAGCCTAAAGGTGGCACTGAGATGTTCTGCCATCCGCGAACAGGTAAGCCACTACCTAAATATCCTCGCATTAAGACACCTAAAGTTGGTGGTATCTTTAAGAAGCCTAAGAACAAGGCACAGCGAGAAGGCCGTGAGCCTTGCGAACTTGATACCCGCGAGTACGTTGCTGGTGCTCCTTACACCCCAGTTGAACATGTTGTGTTTAACCCTTCGtctc                          >  1:235627/1‑301 (MQ=255)`  
                                                                                                                                                                                                                                                                                `gagacaggTTGTACGTAGAGTTAGCTTCTCGCCGCTCTGAGTTGCTCCGTAAATTGACCGAAACGTTCGGCTCGTGGTATCAGCCTAAAGGTGGCACTGAGATGTTCTGCCATCCGCGAACAGGTAAGCCACTACCTAAATACCCTCGCATTAAGACACCTAAAGTTGGTGGTATCTTTAAGAAGCCTAAGAACAAGGCACAGCGAGAAGGCCGTGAGCCTTGCGAACTTGATACCCGCGAGTACGTTGCTGGTGCTCCTTACACCCCAGTTGAACATGTTGTGTTTAACCCTTCGTCTCg                         <  1:370016/294‑1 (MQ=255)`  
                                                                                                                                                                                                                                                                                     `gagTTGTACGTAGAGTTAGCTTCTCGCCGCTCTGAGTTGCTCCGTAAATTGACCGAAACGTTCGGCTCGTGGTATCAGCCTAAAGTTGGCACTGAGATGTTCTGCCATCCGCGAACAGGTAAGCCACTACCTAAATACCCTCGCATTAAGCCACCTAAAGTTGGTGGTCTCTTTAAGAAGCCTAAGAACAAGTCCCAGCGAGAAGGCCGTGAGCCTTGCGAACTTGATACCCGCGAGTACGTTGCTGGTGCTCCTTACACCCCAGTTGAACATGTTGTGTTTTCCCCTTCGTCTCGTGAcc                    >  1:204590/1‑301 (MQ=255)`  
                                                                                                                                                                                                                                                                                        `aagagacaggagTTAGCTTCTCGCCGCTCTGAGTTGCTCCGTAAATTGACCGAAACGTTCGGCTCGTGGTATCAGCCTAAAGGTGGCACTGAGATGTTCTGCCATCCGCGAACAGGTAAGCCACTACCTAAATACCCTCGCATTAAGACACCTAAAGTTGGTGGTATCTTTAAGAAGCCTAAGAACAAGGCACAGCGAGAAGGCCGTGAGCCTTGCGAACTTGATACCCGCGAGTACGTTGCTGGTGCTCCTTACACCCCAGTTGAACATGTTGTGTTTAACCCTTCGTCTCGTGACcaca                 <  1:613288/292‑1 (MQ=255)`  
                                                                                                                                                                                                                                                                                         `tGTACGTAGAGTTAGCTTCTCGCCGCTCTGAGTTGCTCCGTAAATTGACCGAAACGTTCGGCTCGTGGTATCAGCCTAAAGGTGGCACTGAGATGTTCTGCCATCCGCGAACAGGTAAGCCACTACCTAAATACCCTCGCATTAAGACACCTAAAGTTGGTGGTATCTTTAAGAAGCCTAAGAACAAGGCACAGCGAGAAGGCCGTGAGCCTTGCGAACTTGATACCCGCGAGTACGTTGCTGGTGCTCCTTACACCCCAGTTGAACATGTTGTGTTTAACCCTTCGTCTCGTGACcacct                >  1:568810/1‑299 (MQ=255)`  
                                                                                                                                                                                                                                                                                           `tACGTAGAGTTAGCTTCTCGCCGCTCTGAGTTGCTCCGTAAATTGACCGAAACGTTCGGCTCGTTGTATCAGCCTAAAGGTGGCACTGAGATGTTCTGTCATCCGCGAACAGGTAAGCCACTACCTAAATACCCTCGCATTAAGACACCTAAAGTTGGTGGTATCTTTAAGAAGCCTAAGAACAAGGCACAGCGAGAAGGCCGTGAGCCTTGCGAACTTGATACCCGCGAGTACGTTGCTGGTGCTCCTTACACCCCAGTTGAACATGTTGTGTTTAACCCTTCGTCTCGTGACCACATTc              <  1:404435/301‑1 (MQ=255)`  
                                                                                                                                                                                                                                                                                            `aCGTAGAGTTAGCTTCTCGCCGCTCTGAGTTGCTCCGTAAATTGACCGAAACGTTCGGCTCGTGGTATCAGCCTAAAGGTGGCACTGAGATGTTCTGCCATCCGCGAACAGGTAAGCCACTACCTAAATACCCTCGCATTAAGACACCTAAAGTTGGTGGTATCTTTAAGAAGCCTAAGAACAAGGCACAGCGAGAAGGCCGTGAGCCTTGCGAACTTGATACCCGCGAGTACGTTGCTGGTGCTCCTTACACCCCAGTTGAACATGTTGTGTTTAACCCTTCGTCTCGTGACCACATTCa             >  1:187389/1‑301 (MQ=255)`  
                                                                                                                                                                                                                                                                                                   `gTTAGCTTCTCGCCGCTCTGAGTTGCTCCGTAAATTGACCGAAACGTTCGGCTCGTGGTATCAGCCTAAAGGTGGCACTGAGATGTTCTGCCATCCGCGAACAGGTAAGCCACTACCTAAATACCCTCGAATTAAGACACCTAAAGTTGGTGGTATCTTTAAGAAGCCTAAGAACAAGGCACAGCGAGAAGGCCGTGAGCCTTGCGAACTTGATACCCGCGAGTACGTTGCTGGTGCTACTTACACCCCATTTAAACATGTTGTGTTTAACCCTTCGTCTCGTGACCACATTCCGAAGaaa      >  1:470787/1‑301 (MQ=255)`  

                                                                                                                                                                                                                                                                                                          `|`                                                                                                                                                                                                                                                                                                           
`AGAGCAGGGTGAAGAATACGTTGACGGAATGGAGTGGTGGAACTTCAACGAAGAGATGATGGACTATAACGTTCAGGACGTTGTGGTAACTAAAGCTCTCCTTGAGAAGCTACTCTCTGACAAACATTACTTCCCTCCTGAGATTGACTTTACGGACGTAGGATACACTACGTTCTGGTCAGAATCCCTTGAGGCCGTTGACATTGAACATCGTGCTGCATGGCTGCTCGCTAAACAAGAGCGCAACGGGTTCCCGTTTGACACAAAAGCAATCGAAGAGTTGTACGTAGAGTTAGCTGCTCGCCGCTCTGAGTTGCTCCGTAAATTGACCGAAACGTTCGGCTCGTGGTATCAGCCTAAAGGTGGCACTGAGATGTTCTGCCATCCGCGAACAGGTAAGCCACTACCTAAATACCCTCGCATTAAGACACCTAAAGTTGGTGGTATCTTTAAGAAGCCTAAGAACAAGGCACAGCGAGAAGGCCGTGAGCCTTGCGAACTTGATACCCGCGAGTACGTTGCTGGTGCTCCTTACACCCCAGTTGAACATGTTGTGTTTAACCCTTCGTCTCGTGACCACATTCAGAAGAAACTCC  >  NC_001604.1/14796‑15391`

  

Alignment Legend

Aligned base mismatch/match (shaded by quality score): `ATCG/ATCG` `< 3 ≤` `ATCG/ATCG` `< 9 ≤` `ATCG/ATCG` `< 20 ≤` `ATCG/ATCG` `< 34 ≤` `ATCG/ATCG` `< 38 ≤` `ATCG/ATCG`

Unaligned base: `atcg`    Masked matching base: `atcg`    Alignment gap: `‑`    Deleted base: `‑`
